# The use of complementary and alternative medicines among patients with locally advanced breast cancer – a descriptive study

**DOI:** 10.1186/1471-2407-6-39

**Published:** 2006-02-21

**Authors:** Lucy K Helyer, Stephen Chin, Betty K Chui, Barbara Fitzgerald, Sunil Verma, Eileen Rakovitch, George Dranitsaris, Mark Clemons

**Affiliations:** 1 Department of Surgical Oncology, Toronto-Sunnybrook Regional Cancer Centre, Sunnybrook and Women's College Health Sciences Centre, 2075 Bayview Ave, Toronto, ON, M4N 3M5, Canada; 2 Division of Medical Oncology, Toronto-Sunnybrook Regional Cancer Centre, Sunnybrook and Women's College Health Sciences Centre, 2075 Bayview Ave, Toronto, ON, M4N 3M5, Canada; 3 Department of Radiation Oncology, University of Toronto, Cancer Care, Ontario, canada; 4 Department of Radiation Oncology, University of Toronto, Cancer Care Ontario, 600 University Ave Toronto, ON Canada

## Abstract

**Background:**

Complementary and alternative medicine (CAM) use is common among cancer patients. This paper reviews the use of CAM in a series of patients with locally advanced breast cancer (LABC).

**Methods:**

Women with LABC attending a specialist clinic at a single Canadian cancer centre were identified and approached. Participants completed a self-administered survey regarding CAM usage, beliefs associated with CAM usage, views of their risks of developing recurrent cancer and of dying of breast cancer. Responses were scored and compared between CAM users and non-users.

**Results:**

Thirty-six patients were approached, 32 completed the questionnaire (response rate 89%). Forty-seven percent of LABC patients were identified as CAM users. CAM users were more likely to be younger, married, in a higher socioeconomic class and of Asian ethnicity than non-users. CAM users were likely to use multiple modalities simultaneously (median 4) with vitamins being the most popular (60%). Motivation for CAM therapy was described as, "assisting their body to heal" (75%), to 'boost the immune system' (56%) and to "give a feeling of control with respect to their treatment" (56%). CAM therapy was used concurrently with conventional treatment in 88% of cases, however, 12% of patients felt that CAM could replace their conventional therapy. Psychological evaluation suggests CAM users perceived their risk of dying of breast cancer was similar to that of the non-Cam group (33% vs. 35%), however the CAM group had less severe anxiety and depression.

**Conclusion:**

The motivation, objectives and benefits of CAM therapy in a selected population of women with LABC are similar to those reported for women diagnosed with early stage breast cancer. CAM users display less anxiety and depression and are less likely to believe they will die of their breast cancer. However the actual benefit to overall and disease free survival has yet to be demonstrated, as well as the possible interactions with conventional therapy. Consequently more research is needed in this ever-growing field.

## Background

Cancer patients take a wide range of complementary and alternative medicines (CAM). These include ingested therapies such as herbs, and vitamins, homeopathic remedies and traditional Chinese medicines as well as psychological, physical and spiritual techniques [1-4]. Currently between 20% and 84% of cancer patients are using CAM therapies [1-6]. Predisposing characteristics for CAM use have been shown to include a high level of education, high-income bracket, ethnicity, marital status, adjuvant therapy and presence of anxiety or depression [1-3,7-9].

While no data reports improved survival or disease free interval with the use of CAM [10], many cancer patients attribute CAM therapy with providing them a feeling of control over their disease. CAM therapies are also believed to have properties such as, 'boosting' the immune system and preventing/treating cancer [2,11]. Studies comparing CAM use in women with advanced stage cancer to those with early stage cancer find goals of therapy to be similar, therapeutic and psychological [11]: i.e. to relieve symptoms [2,5,9,11], to exude greater control over their life and express hopefulness over there disease and life [7].

Patients with locally advanced breast cancer (LABC) are a unique population. They have tumours that are frequently large, involve the regional lymph nodes or extend beyond the breast tissue [12]. Even with aggressive therapy the 5-year survival rate for these patients is poor at around 55% [13].

We recently reported in a population of women with breast cancer including all stages of disease, that CAM users are more likely to be younger, better educated and in full time employment compared to non-users [14]. In addition while there was no difference between CAM usage with respect to tumour stage, CAM therapy was associated with an increased perception of breast cancer recurrence and of breast cancer-related death [14].

The objective of this study was to identify the overall usage of CAM in patients with LABC, determine their motivation and objectives for usage. Secondarily the effect of CAM therapy on patient's beliefs and perceptions of their disease was examined.

## Methods

The breast cancer site at the Toronto-Sunnybrook Regional Cancer Centre (TSRCC) has Canada's only specialist multidisciplinary clinic for women with LABC. Between June 2003 and June 2004 consecutive outpatients with a diagnosis of breast cancer were screened for eligibility. Only patients with locally advanced breast cancer were included in this study. Patients were invited to participate in this study examining the prevalence of CAM therapy by a study nurse prior to their visit with their oncologist. All surveys were completed under the supervision of a trained data manager in a private room, and any questions or concerns were addressed by their own oncologist (ER or MC). Patients were reassured that participation in this study in no way altered there medical care. Patients were excluded if they did not have English proficiency or did not give informed consent. Independent confirmation of diagnosis, stage and treatment in all patients was performed by one of the investigators (ER or MC). Study approval was obtained from the local Research and Ethics Board.

The self-administered questionnaire (appendix 1) was developed to obtain information on demographics, conventional therapy and CAM use [14]. Sixteen of the most common reasons for using CAM and patient perceptions of CAM therapy were derived from the current literature and listed as choices [2]. Patient's beliefs and perceptions of their disease were assed using a Likert scale with larger scores representing higher perceptions of risk [14]. Beliefs were scored on a scale ranging from 1(strongly disagree) to 5 (strongly agree), while perceptions were scored on a scale from very unlikely (-2) to highly likely (+2).

The Hospital Anxiety and Depression scale was used to assess psychological distress [15]. This instrument gives separate measures of anxiety and depression with seven items for anxiety and seven items for depression (range of scores from 0 to 21). Each item (anxiety and depression) is scored separately. Scores <7 represent a non-case, scores 8–10 represent a possible case and those above 10 represent significant disease related to anxiety or depression. The sensitivity of this test has been reported to be 74% with a specificity of 75% [16].

Due to the small number of patients the results of this study are presented descriptively as means, medians and proportions. Statistical examination was unable to show significant difference between the CAM users and Non CAM users due to small sample size and inadequate statistical power. Use of comparative tests would have increased the probability of a type I error.

## Results

### Demographics

Thirty-two of the 36 patients approached completed the questionnaire (response rate of 89%). Patient demographics are outlined in Table 1. All patients were female, mean age of 54 (range 29–91 years). Fifteen of the 32 patients (47%) were identified as CAM users. The CAM users tended to be younger (mean 49 years vs. 59 years) and 86% were in a current relationship versus 52% of non-Cam users. Over 80% of both groups had completed high school, while 30% in the non-CAM users had a graduate degree compared with 13% in the CAM group. The numbers of patients remaining at work, on sick leave, or at home were comparable as was income distribution between groups. The diverse ethnic population reflects our patient population.

### Conventional therapy

The CAM user group was more likely to have had more intensive therapy for breast cancer, 30% of patients in the CAM group had all three modalities of treatment; surgery, chemotherapy and radiation in comparison with 14% in the non CAM user group (Table 1). The use of hormonal therapy was equal between the groups. The presence of private insurance and a family physician was also more common in those using CAM techniques (66% vs. 58%) and (100% vs. 88%) respectively. CAM users were also more likely to be involved in cancer support groups (20% vs. 5%) and if not, more interested in joining one than their counter parts (25% vs. 13%).

### Complementary medicine

The use of complementary alternative medicine was divided into dietary, herbal-homeopathy, psychological and physical methods (Table 2). Many patients used more than one modality with a mean of 4, (range 1–11). Forty percent of the CAM group had used these techniques prior to their diagnosis of breast cancer and all continued throughout therapy. The most common source of information about complementary medicine was a conventional physician (40%) followed by pharmacist and friend (20%). Two thirds of patients (10) researched CAM therapy prior to starting, 5 patients paid for consultation with prices ranging from $60 to $200 CDN. The cost of CAM varied between patients with the majority spending up to $50 CDN/month (46%) some however spending over $100 CDN/month (23%).

The most common form of CAM was vitamin therapy, and over 60% of CAM users had taken supplements and additives. Forty percent of the patients using CAM were on restrictive diets; either low in fat, vegetarian or using soy products. Two thirds of patients used psychological techniques including faith and spiritual healing (40%) and guided imagery (26%). 86% of patients felt their CAM was helpful and beneficial for their disease. Sixty four percent of patients using CAM had informed their oncologist and believed it was important information for their oncologist to know.

### Beliefs associated with CAM

Patients' reasons and beliefs regarding CAM therapy were explored asking questions phrased, "Do you believed CAM......?"(Table 3). The most common reason for CAM use in this population was "to assist the body's natural forces to heal"(75%). Other common reasons were: to assist other treatments (62%), relieve symptoms (62%), and increase quality of life (62%). 56% felt there would not be any side effects and 43% believed that CAM was perfectly safe with no adverse effects or interactions with other cancer therapies. 12.5% of patients felt that CAM therapy would make them less likely to accept conventional treatments and 18% believed it would reduce the chance that other therapies would work. Two patients refused conventional treatment, believing the CAM therapy would cure their breast cancer. One quarter of patients believed CAM therapy would prevent the spread of their disease and 18% believed it would cure the cancer. Half of respondents were indifferent with respect to the belief that CAM therapy would cure their cancer, prevent its spread and/or prevent a recurrence.

### Perceived risk of breast cancer and associated anxiety and depression

The risk assessment asked four questions to show patients' perception of developing and dying of recurrent breast cancer. CAM users believed they would more likely die of something other than their breast cancer (72% vs 42%) and their cancer was less likely to recur elsewhere after treatment (53% vs 35%). However over 60% of all patients with LABC (CAM users and non-users) felt there disease would not recur in the treated breast or cause their death (Table 4).

**Figure 1:**
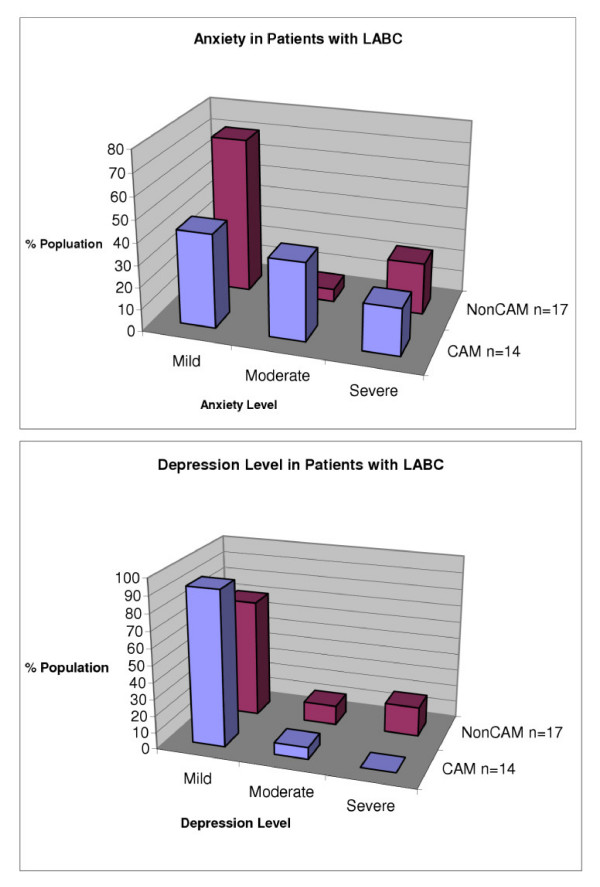
Anxiety and Depression Distribution Among Patients with LABC.

The Hospital Anxiety and Depression Scale (HADS) [15] (figure 1) was included to demonstrate the psychological health of the patient population. Anxiety was seen in both groups CAM user and non-CAM user. Those patients using CAM therapy has less severe anxiety. The highest anxiety scores in the CAM group were recorded in patients using greater than four modalities of CAM. CAM users' described their depression as mild more frequently in comparison to the non-CAM users.

## Discussion

Depending on the population studied, the prevalence of CAM use among breast cancer patients is 20%–84% [1-6]. Forty seven percent of patients with LABC at our institution were CAM users, similar to the proportion of users in a non-stage specific group at our institution [14]. Variance in degree of use has been attributed to socio-demographic and clinical differences in study populations more so than stage of disease.

Women with LABC are a unique population. They present to medical attention with breast cancer at an advanced stage and they therefore have a poorer prognosis [17]. Reasons proposed for presentation with advanced disease depends largely on the population studied and may include the biology of the tumour, unwillingness of the patient to consult medical attention due to fear, denial [18] or pursuit of alternative therapy [19-21], or lack of patient education [22] but is largely unknown. Most patients, as in our study, use CAM therapy as an adjunct to conventional treatment [2,5]. Only 12% of our patients refused conventional treatment preferring CAM alternatives. Despite widespread use of CAM therapy among cancer patients, the efficacy in the course of cancer therapy has not been substantially confirmed [10,18]. Jacobson *et al*. reviewed English language publications from 1980 to 1997, looking for supportive evidence for CAM therapy and although most studies lacked vigorous scientific design, there was no definite survival benefit with the addition of CAM therapy [10]. Physical methods such as acupuncture, massage and mind-body techniques have modest evidence to show patient benefit with respect to anxiety, depression and quality of life [23,24]. The use of CAM therapies has also been shown to correlate with patients coping styles and may fulfil an important psychological need in some patients [25].

The reasons patients give for using CAM are varied, but many concentrate around feelings of control over therapy or of aiding the body over come disease and harmful effects of traditional therapy [4,26,27]. Most CAM techniques and substances are felt to be innocuous, as most patients associate 'natural' therapy with safe and non-toxic therapy, a view supported by many health food stores [19]. Side effects and adverse effects are consequently minimized and as a result, poorly communicated to consumers. This perception of 'natural' may encourage patients to take CAM to the exclusion of the conventional therapies with the multiple documented side effects and adverse events [19]. Indeed the global consumption of these therapies continues to rise [9].

Our patient population using CAM was similar to others reported; all woman, in a higher income bracket, currently in a relationship, and more likely to join or be part of a support group [2,14,25,28,29]. However, the non-CAM users in our study were more likely to have graduate degrees and thus higher education. This inverse relationship between CAM use and education has been previously described by Dy *et al*. where participants in phase I trials were found to be less likely to take pharmacologic CAM if they had attained a high level of formal education[30]. However, the majority of evidence supports a higher use of CAM therapy in those with higher socioeconomic and education levels [2,5,9,25,28,29]. Ethnicity has also been found to be a CAM stratifying characteristic [26,29]. Although we have a small sample size, those of Asian heritage were more likely to pursue CAM therapy. This may emphasize the importance of community and societal support on patient treatment decisions and ultimately cultural acceptance of traditional medicine.

CAM users in our study were more likely to have the support of a partner, family doctor, and be in or interested in joining a support group. This difference in amount of support whether from a partner, family doctor or group delineates our population in a dramatic fashion. The participation in a support group is recognized as highly predictive of CAM use, why is not known but maybe intrinsic to the patients joining the group or as an end result of group interaction [2,21,29,31].

Thirty percent of our patients in the CAM group compared with 14% in the non-CAM group had combination treatment with surgery, chemotherapy and radiation. Similarly, the literature reflects, the more strenuous the treatment the more likely patients are to use CAM [5,17]. Most women used CAM as an adjunct to this intensive therapy, 75% believe that it helped with symptom control, assisted traditional treatments, boosts the immune system and increased the quality of life, while attributing it little to no curative properties. The majority (86%) of our patients taking CAM believed that it helped and were positive about their experience. However, two women in our study refused mainstream treatment and were persisting with primary CAM therapy. Due to the questionnaire form we were unable to better characterize this segment of our population.

Earlier studies showed patients using CAM had higher levels of stress, exhibited more severe anxiety and worse depression[3]. These patients consequently had a worse quality of life. As we have previously published, the CAM users in an unselected population of breast cancer patients exhibited higher levels of anxiety and depression over the non-users[14]. However, our LABC patients, in actuality seemed to be suffering less severe anxiety and felt their mood to be higher (Figure 1). This maybe explained by Moshen et al., who postulated there are two subgroups of patients, one group who actively copes with stress and another who has considerable adjustment problems [25]. The latter group with adjustment problems were more likely to use greater than four CAM therapies and express a depressive coping behaviour. The five patients who used greater than four CAM therapies in our study described a greater level of anxiety but not depression.

Anxiety is a feeling centred on fear and involves worry, apprehension and dread. Those patients with strong beliefs in CAM therapy are able to lessen these feelings, while those who are 'non-believers' are unable assuage this anxiety. Depression meanwhile, is associated with feelings of sadness and hopelessness; CAM therapy provides a positive influence on conventional treatment and may moderate these thoughts. Differences between CAM users and nonusers with respect to anxiety and depression maybe difficult to show in patients with advanced cancers i.e. LABC, and may explain the difference between the non selected and LABC population.

Our studies primary limitation is sample size, and although our breast centre specializes in treatment of locally advanced breast cancer; with increased screening and education this is becoming a rare diagnosis. Consequently statistical evaluation of the data was unable to be performed and instead a descriptive study was chosen. Small sample size may also account for the discrepancy seen in non-CAM users having a higher level of education. Patient selection in an oncology clinic may have overlooked patients avoiding conventional medicine for treatment of their breast cancer and selection bias may be present. In conclusion, due to the fact this is a single institution study, where the diagnosis and treatment of LABC is a priority the results may not be comparable to other non-selected populations of breast cancer patients.

## Conclusion

Studies are needed to determine the mechanism of action of many of the popular additives, and thus elucidate the side effects and adverse effects. Certainly, CAM therapies are used in part as coping mechanisms by many patients. However the benefits or drawback of this type of therapy is poorly understood, consequently more education and research aimed at both physician and patient is necessary to elucidate the complex psychotherapeutic effect of many of these techniques.

## Abbreviations

CAM complementary alternative medicine

LABC locally advanced breast cancer

## Competing interests

The author(s) declare that they have no competing interests.

## Authors' contributions

LKH – final analysis and drafted the manuscript

BC collected data

SC collected data and did initial analysis

MC conceived the study, participated in its design and coordination and helped draft the manuscript

BF, SV, GD reviewed the manuscript.

All authors read and approved the final manuscript

## Appendix 1

Questionnaire given to women with LABC about their CAM use.

## Pre-publication history

The pre-publication history for this paper can be accessed here:



## Figures and Tables

**Table 1:** Demographics of the LABC population

	All LABC Patients n = 32	CAM Patients n = 15	Non-CAM Patients n = 17
Mean Age (years)	54	49	59
Range	29 to 91	29 to 73	34 to 91

**Ethnic Background**			

African-Canadian	9%	0%	17%
Asian	9%	13%	5%
Caucasian	46%	40%	52%
Jewish	9%	20%	0%
Middle Eastern	3%	6%	0%
Mixed Ethnicity	3%	0%	5%
Unknown	6%	13%	0%

**Marital Status**			

Married/Common Law	68%	86%	52%
Widowed/Divorced	12%	0%	23%
Single	18%	13%	23%

**Education:**			

Did not completed	12%	13%	11%
Completed high school	34%	33%	35%
Completed university/college	31%	40%	23%
Completed Post Graduate Degree	21%	13%	29%

**Employment Status**			

Full time	28%	26%	29%
Part time	6%	13%	0%
Unemployed/homemaker	31%	20%	41%
Sick Leave	34%	40%	29%

**Household Income (10,000 CND)**			

0–30	25%	33%	18%
31–70	41%	33%	50%
71–100	9%	13%	6%
>100	22%	20%	25%

**Breast Cancer Therapies**			

Surgery	40%	46%	35%
Chemotherapy	81%	93%	70%
Radiation Therapy	25%	33%	17%
Hormonal Therapy	37%	40%	35%

**Table 2:** CAM use among LABC patients (n = 15)

**Mode of CAM Use:**	**% Population (n = 15)**
**Dietary**	

Macrobiotic	13
Vitamin therapy	66
Megavitamins	20
Minerals	40
Low-fat or vegetarian diets	40
Soy Products	26

**Herbal/Homeopathy**	20

**Psychological Methods**	
Meditation	10
Guided Imagery	26
Hypnosis	6
Faith and Spiritual	40

**Physical Methods**	
Massage therapy	26
Acupuncture	6
Acupressure	6
Yoga	26
Tai Chi	13

**Alternative Providers:**	
Chiropractor	6
Naturopathic Doctor	6
Acupuncturist	6
TCM Doctor	6
Others	13

**Table 3:** Beliefs and perceptions of CAM therapy (n = 15).

**Beliefs**	**Percentages**		
	
**I believe CAM therapy will**	Unlikely (1,2)	Indifferent (3)	Likely (4,5)
Cure Cancer	31.3%	**50.0%**	18.8%
Prevent Spread	25.0%	**50.0%**	25.0%
Assist other treatments	25.0%	12.5%	**62.5%**
Relieve symptoms	18.8%	18.8%	**62.5%**
Have side effects	**56.3%**	12.5%	31.3%
Weaken body's natural reserves	**62.5%**	18.8%	18.8%
Patient's Fault if they don't work	**75.0%**	18.8%	6.3%
Assist the body's natural forces to heal	6.3%	18.8%	**75.0%**
Easy to understand how they work	**37.5%**	25.0%	**37.5%**
Boost to Immune System	18.8%	25.0%	**56.3%**
Perfectly safe	25.0%	31.3%	**43.8%**
Increase Quality of Life	6.3%	31.3%	**62.5%**
Feeling of control over the cancer	25.0%	18.8%	**56.3%**
Prevent recurrence of Cancer	18.8%	**56.3%**	25.0%
Reduce the chance that other therapies will work	37.5%	43.8%	18.8%
Makes them less likely to accept conventional treatments	**50.0%**	37.5%	12.5%

**Table 4:** Risk perception in LABC patients

**Risk Perception** How likely is it that....?	Non CAM Users % people who rated (n = 17)	CAM Users % people who rated (n = 15)
**Cancer recurrence in same breast**		
Unlikely	53 (9)	57 (8)
Likely	47 (8)	43 (7)
**Cancer recurrence elsewhere**		
Unlikely	35 (6)	53 (8)
Likely	65 (11)	47 (7)
**Likely die of cancer**		
Unlikely	65 (11)	67 (10)
Likely	35(6)	33.(5)
**Likely die of something else**		
Unlikely	58 (10)	27 (4)
Likely	42 (7)	73 (11)

## References

[B1] Eisenberg DM, Kessler RC, Forster C, Norlock FE, Calkins DR, Deldano TL (1993). Unconventional medicine in the United States; prevalence, costs and patterns of use. N Engl J Med.

[B2] Boon H, Stewart M, Kennard MA, Sawka C, Brown JB, McWilliam C, Gavin A, Baron RA, Aaron D, Haines-Kamka T (2000). The use of complementary/alternative medicine by breast cancer survivors in Ontario: Prevalence and perceptions. J Clin Oncol.

[B3] Burstein HJ, Gelber S, Guadagnoli E, Weeks J (1999). Use of alternative medicine by women with early-stage breast cancer. N Engl J Med.

[B4] Ashikage T, Bosompra K, O'Brien P, Nelson L (2002). Use of complementary and alternative medicine by breast cancer patients: prevalence, patterns and communication with physicians. Supp Care Can.

[B5] Richardson MA, Sanders T, Palmer JL, Greisinger A, Singletary SE (2000). Complementary/alternative medicine use in a comprehensive cancer centre and the implications of oncology. J Clin Oncol.

[B6] Ernst E, Casselith BR (1998). The prevalence of complementary/alternative medicine in cancer: a systematic review. Cancer.

[B7] Astin JA (1998). Why patients use alternative medicine: Results of a national study. JAMA.

[B8] Crocetti E, Crotti N, Feltrin A, Ponton P, Geddes M, Buiatti (1998). The use of complementary therapies by breast cancer patients attending conventional treatment. Eur J Can.

[B9] Rees RW, Feigel I, Vickers A, Zollman C, McGurk R, Smith C (2000). Prevalence of complementary therapy use by women with breast cancer: a population – based study. E J Cancer.

[B10] Jacobson JS, Workman S, Kronenberg F (2000). Research on complementary/alternative medicine for patients with breast cancer: a review of the biomedical literature. J Clin Oncol.

[B11] Shen J, Andersen R, Albert PS, Wenger N, Glaspy J, Cole M, Shekelle P (2002). Use of complementary/alternative therapies by women with advanced stage breast cancer. BMC Complementary and Alternative Medicine.

[B12] Singletary SE, Allred C, Ashley P, Bassett W, Berry D, Bland KI, Borgen PI, Clark G, Edge SB, Hayes DF, Hutter RVP, Morrow M, Page DL, Recht A, Theriault RL, Thor A, Weaver DL, Wieand S, Green FL (2002). Revision of the American Joint Committee on Cancer staging system for breast cancer. J Clin Oncol.

[B13] Therasse P, Mauriac L, Welnicka-Jaskiewicz M, Bruning P, Cufer T, Bonnefoi H, Tomiak E, Pritchard KI, Hamilton A, Piccart MJ, EORTC (2003). Final results of a randomized phase III trial comparing cyclophosphamide, epirubicin, and fluorouracil with a dose-intensified epirubicin and cyclophosphamide + filgrastim as neoadjuvant treatment in locally advanced breast cancer: an EORTC-NCIC-SAKK multicenter study. J Clin Oncol.

[B14] Rakovitch E, Pignol J, Chartier C, Ezer M, Verma S, Dranitsaris G, Clemons M (2005). Complementary and alternative medicine use is associated with an increased perception of breast cancer risk and death. Breast Cancer Research and Treatment.

[B15] Zigmond AS, Snaith RP (1983). The hospital anxiety and depression scale. Acta Psychiatrica Scandinavica.

[B16] Hopwood P, Howell A, Maguire P (1991). Screening for psychiatric morbidity in patients with advanced breast cancer: validation of two self-report questionnaires. Br J Cancer.

[B17] Richards MA, Smith P, Ramirez AJ, Fentiman IS, Rubens RD (1999). The influence on survival of delay in the presentation and treatment of symptomatic breast cancer. B J C.

[B18] Malik IA, Gopalan S (2003). Use of CAM results in delay in seeking medical advice for breast cancer. Eur J Epidemiol.

[B19] Mills E, Ernst E, Singh R, Ross C, Wilson K (2003). Health food store recommendations: implications for breast cancer patients. Breast Cancer Res.

[B20] Hisham AN, Yip CH (2004). Overview of breast cancer in Malaysian women: A problem with late diagnosis. Asian J Surg.

[B21] Mohamed I, Williams K, Tamburrino M, Wryobeck J, Carter S (2005). Understanding locally advanced breast cancer: what influences a woman's decision to delay treatment?. Prev Med.

[B22] Ramirez A, Westcombe A, Burgess C, Sutton S, Littlejohns P, Richards M (1999). Factors predicting delayed presentation of symptomatic breast cancer: a systematic review. Lancet.

[B23] Deng G, Cassileth B (2005). Integrative oncology: complementary therapies for pain, anxiety and mood disturbance. CA Cancer J Clin.

[B24] Anon (1996). Integration of behavioural and relaxation approaches into the treatment of chronic pain and insomnia. NIH technology assessment panel on integration on behavioral and relaxation approaches into the treatment of chronic pain and insomnia. JAMA.

[B25] Moschen R, Kemmler G, Schweigkofler H, Holzner B, Dunser M, Richter R, Fleischhacker WW, Sperner-Unterweger B (2001). Use of alternative/complementary therapy in breast cancer patients a psychological perspective. Support Care Cancer.

[B26] Cui Y, Shu X, Gao Y, Wen W, Ruan Z, Jin F, Zheng W (2004). Use of complementary and alternative medicine by Chinese women with breast cancer. Breast Cancer Research and Treatments.

[B27] Patterson R, Neuhouser ML, Hedderson MM, Schwartz SM, Standish LJ, Bowen DJ, Marshall LM (2004). Types of alternative medicine used by patients with breast cancer, or prostate cancer: predictors, motives and costs. J Altern and Complement Med.

[B28] Navo MA, Phan J, Vaughan C, Palmer JL, Michaud L, Jones KL, Bodurka DC, Basen-engquist K, Hortobagyi GN, Kavanagh JJ, Smith JA (2004). An assessment of the utilization of complementary and alternative medication in women with gynecologic or breast malignancies. J Clin Oncol.

[B29] Lee M, Lin S, Wrensch M, Adler S, Eisenberg D (2000). Alternative therapies used by women with breast cancer in four ethnic populations. J Natl Cancer Inst.

[B30] Dy GK, Bekele L, Hanson LJ, Furth A, Mandrekar S, Sloan JA, Adjei AA (2004). Complementary and alternative medicine use by patients enrolled onto phase I clinical trials. J Clin Oncol.

[B31] Nagel G, Hoyer H, Katenkamp D (2004). Use of complementary and alternative medicine by patients with breast Cancer: observations from a health survey. Supp Care Cancer.

